# Gene therapy approaches to restore retinal cGMP channel function

**DOI:** 10.1186/2050-6511-16-S1-A9

**Published:** 2015-09-02

**Authors:** Martin Biel

**Affiliations:** 1 Center for Integrated Protein Science Munich CiPSM at the Department of Pharmacy, Center for Drug Research, Ludwig-Maximilians-Universität München, Butenandtstraße 5-13, D-81377 München, Germany

## 

Vertebrate vision is based on light-mediated alterations of the cyclic GMP concentration in outer segments of retinal photoreceptors. These chemical signals are converted by cyclic nucleotide-gated (CNG) channels into electrical signals that are further processed within the retina and, finally, the visual cortex of the brain. Dysfunctional cGMP signaling is associated with visual impairment and has been identified in several types of inherited blinding diseases. In particular, loss-of-function mutations in CNG channel subunits expressed in cone and rod photoreceptors were identified to cause achromatopsia (total color blindness) and retinitis pigmentosa, respectively. Besides displaying functional defects, the pathologies of both diseases are characterized by retinal degeneration that may be triggered by profound alterations in the cGMP and Ca2+ homeostasis. Our laboratory has generated genetic mouse models to dissect the molecular pathways underlying the multifaceted pathomechanisms of achromatopsia and retinitis pigmentosa. Complementary to these studies viral expression vectors based on AAVs (adeno associated virus) have been developed to rescue vision in genetic mouse models. These gene therapy approaches have now evolved to a point that allows clinical application in human patients in the near future. Our current approaches in this exciting new field will be discussed.

